# An Unusual Case of Posterior Reversible Encephalopathy Syndrome

**DOI:** 10.5811/cpcem.2017.3.30999

**Published:** 2017-07-14

**Authors:** Robert P. Zemple, Tomer Pelleg, Moises R. Cossio

**Affiliations:** * Carilion Clinic, Department of Emergency Medicine, Roanoke, Virginia; † Carilion Clinic, Department of Internal Medicine, Roanoke, Virginia; ‡ Carilion Clinic, Department of Pulmonary Critical Care, Roanoke, Virginia

## Abstract

A 21-year-old pregnant female with no significant past medical history presented with acute onset headache and nausea as well as tonic-clonic seizures, then rapidly decompensated into a coma with complete absence of brainstem reflexes. The patient was ultimately diagnosed with hemolysis, elevated liver enzymes, and low platelets (HELLP syndrome) and subsequent posterior reversible encephalopathy syndrome (PRES) with brainstem involvement. Emergent delivery and blood pressure control resulted in rapid and complete neurologic recovery.

## INTRODUCTION

Posterior reversible encephalopathy syndrome (PRES), first described by Hinchey et al in 1996,^1^ is a reversible condition classically associated with headache, altered mental status, seizures, and visual deficits. Vasogenic edema, most commonly manifesting as posterior white matter changes, is frequently appreciated.^2^ Encephalopathy is the most common presenting symptom, which has been described in approximately 92% of patients, followed closely by seizure 75–87%, and headache in 53%. 3 Without immediate management and definitive treatment, significant morbidity and mortality can result.

## CASE REPORT

A 21-year-old female with no significant past medical history presented to an outside hospital accompanied by her father, following two witnessed episodes of generalized tonic-clonic movement. Initial seizure activity was controlled with a single, appropriate dose of intravenous lorazepam; however, she quickly became obtunded and required intubation for airway protection. A bedside ultrasound revealed a previously unknown pregnancy of 32–35 weeks gestation. Basic laboratory evaluation demonstrated a mildly elevated serum creatinine and no other abnormalities. An emergent non-contrast head computed topography (CT) demonstrated findings of diffuse anoxic brain injury. With no signs of neurologic recovery the patient was transferred to a tertiary academic medical center.

Upon arrival to the accepting institution the patient was tachycardic at 129 beats per minute and hypertensive with a blood pressure of 229/158 mm Hg. Initial physical examination revealed an unresponsive and gravid female, intubated and mechanically ventilated. Neurologic exam demonstrated the complete absence of a gag reflex with no active paralytic on board from the outside facility. Her pupils were non-reactive and she had no corneal reflexes; doll’s eyes phenomena were present. Invasive arterial monitoring and central venous access were rapidly acquired. Immediate hypertensive management with a continuous intravenous nicardipine infusion was initiated. The patient also received a concomitant magnesium infusion for seizure control. Complete laboratory analysis demonstrated transaminitis (aspartate aminotransferase [AST] 569 IU/L, alanine aminotransferase [ALT] 159 IU/L), thrombocytopenia (43,000/ml), and anemia, consistent with HELLP syndrome.

The patient underwent emergent cesarean section and a live female infant was delivered. Post-delivery repeat non-contrast head CT (initial at outside hospital) revealed slender lateral ventricles and diffuse cerebral swelling; low-density areas were seen in the parieto-occiptal region as well as indistinct areas of the basal ganglia, putamen and thalamus (Image), suggesting either anoxic brain injury or atypical posterior reversible encephalopathy syndrome.

Following delivery, the patient’s blood pressure continued to improve and the nicardipine infusion was discontinued. Antiepileptic therapy was initiated and the magnesium infusion was gradually tapered. Shortly thereafter, the patient’s brainstem reflexes began to return; six hours post-delivery the patient’s pupils were reactive and she demonstrated voluntary movement of all four extremities. Her neurologic status continued to improve over the next 16 hours and she was liberated from ventilatory support the subsequent morning.

The subsequent neurological examination revealed no cognitive or reflexive deficits; she was able to ambulate and communicate appropriately. Her laboratory data began to improve and she was transferred to the postpartum unit the following day where she continued to recover. On hospital day six, the patient was discharged with a healthy baby girl.

## DISCUSSION

New-onset seizures, in the context of an unknown pregnancy, established the diagnosis of eclampsia in a patient with laboratory-confirmed HELLP syndrome resulting in a distinctly unusual presentation of PRES. PRES-induced brainstem areflexia and coma is a remarkable and unusual entity, representing one of the few rapidly reversible causes of catastrophic neurologic insult. HELLP syndrome has limited and non-specific neurologic findings, often with headache or blurred vision, similar to findings in eclampsia, making the distinction difficult without laboratory or radiographic evaluation.

CPC-EM CAPSULE

What do we already know about this clinical entity?

We know the clinical presentation of PRES, but rarely see complete, rapid resolution of all neurology symptoms in patients presenting comatose without brainstem reflexes.

What makes this presentation of disease reportable?

This case is an unusual presentation of two potentially morbid conditions associated with pregnancy that need aggressive treatment stemming from rapid recognition.

What is the major learning point?

It supports aggressive treatments for this condition, as complete neurologic resolution is possible, as well as the use of other first line antihypertensive agents in pregnant patients.

How might this improve emergency medicine practice?

Further supports the notion in emergency medicine we need to think beyond a single diagnosis to explain all presentations, as occasionally 2 major pathologies present.

This constellation of symptoms represents a neurologic emergency. Imaging in the form of a head CT most commonly reveals the development of vasogenic edema to support the diagnosis. The vasogenic edema is generally found in the posterior white matter of the cerebral hemispheres and is frequently seen in the parieto-occipital lobes bilaterally; however, it has been reported throughout the brain.^4^,^5^ Diffusion-weighted imaging is used to better differentiate between cytotoxic and vasogenic edema.

The neurologic findings associated with PRES can be directly attributed to the vasogenic edema found on head CT. Although the presence of vasogenic edema is well documented with PRES, the inciting pathophysiology remains disputed. Early postulates suggested that intense cerebral autoregulatory vasoconstriction occurs in response to acute elevations in blood pressure. This vasoconstriction leads to decreased cerebral blood flow and resultant ischemia, which in turn causes edema in the watershed arterial regions. More recently, investigators proposed that the marked hypertensive state leads to forced vasodilatation through failure of the cerebral autoregulatory system resulting in hydrostatic extravasations of fluid into the interstitium.^2^,^4^

While the pathophysiology of PRES remains controversial, there is a significant amount of literature detailing the multiple conditions that have been associated with the development of PRES. Most commonly, significant elevations in blood pressure are noted. Certain medications, especially cyclosporine, tacrolimus and other chemotherapeutic agents, have been associated with the development of PRES. Regardless of the etiology, the clinical prognosis is often excellent. Given appropriate supportive intensive care, control of hypertension, and removal of the offending agents, PRES is often completely reversible with minimal lasting neurologic deficits.^6^ In the setting of eclampsia, parturition remains the definitive means to recovery.

## CONCLUSION

PRES in the setting of eclampsia is a well-established occurrence;^5^, ^7^–^10^ however, concomitant HELLP syndrome and coma remains a rare entity. It is unlikely that the appropriate dose of benzodiazepenes to control the seizure onset contributed significantly to her obtunded state at presentation. Moreover, brainstem areflexia represents an extremely poor prognostic factor and may portend brain death. 13 Neurologic recovery in the face of such brain stem dysfunction is a truly exceptional event. It is important to recognize this condition clinically, as CT head imaging demonstrates only 40% correlate to magnetic resonance imaging (gold standard) when making this diagnosis, and CT is the imaging modality of choice in the emergency department.^14^ Particularly interesting is the rapid and full clinical recovery within 30 hours from onset of encephalopathy leading to hospital discharge just six days after the initial presentation.

## Figures and Tables

**Image:**
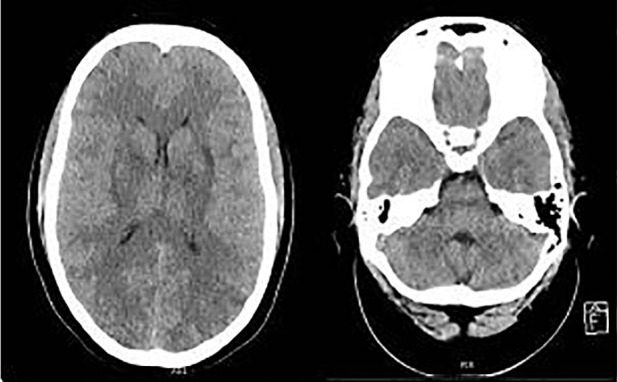
Image of computed topography of head, axial view. **a.** There is no midline shift. The lateral ventricles are extremely slender. This may reflect diffuse cerebral swelling. There are multiple areas of indistinctness surrounding the basal ganglia, putamen, and lateral thalami. **b.** The same findings in ‘a’ can be extended to this more caudal section. Note diffuse indistinctness of the sulci and slender ventricles.
